# Influence of plaque characteristics by coronary computed tomography angiography on lesion-specific ischemia: a systematic review and meta-analysis

**DOI:** 10.1007/s00330-025-11516-1

**Published:** 2025-03-27

**Authors:** Nadia Iraqi, Bjarne L. Nørgaard, Damini Dey, Jawdat Abdulla

**Affiliations:** 1 Department of Clinical Medicine, Health, Aarhus University, Aarhus, Denmark; 2 Department of Cardiology, Aarhus University Hospital, Aarhus, Denmark; 3 Department of Internal Medicine, Regional Hospital, Horsens, Denmark; 4 Biomedical Imaging Research Institute, Cedars-Sinai Medical Center, Los Angeles, CA USA; 5 Department of Cardiology, Amager-Hvidovre University Hospital, Copenhagen, Denmark

**Keywords:** Computed tomography, Atherosclerotic plaque, Fractional flow reserve

## Abstract

**Objectives:**

To evaluate the association between plaque characteristics and burden by coronary computed tomography angiography (CCTA) and ischemia determined by invasively measured fractional flow reserve (FFR), and whether the addition of plaque characteristics improves ischemia discrimination beyond coronary stenosis alone.

**Methods:**

A systematic literature review and meta-analysis of studies from PubMed, EMBASE, and the Cochrane Library databases, published between January 2005 and October 2024 were conducted to assess the relationship between quantitative and qualitative coronary plaque characteristics and invasive FFR. Pooled analyses were performed using weighted mean difference for plaque volumes with 95% confidence intervals and odds ratios for qualitative plaque findings.

**Results:**

A total of 29 studies involving 4416 patients (mean age 63 ± 9 years and 71% male) with predominantly stable coronary artery disease were included. Data on 3923 lesions and 3520 vessels were pooled. Total plaque, non-calcified plaque, and percent aggregate plaque volumes, as well as percent plaque burden, were inversely associated with FFR at both per-lesion and per-vessel levels (all, *p*-values < 0.05). The presence of high-risk plaque characteristics, including low-attenuation plaque, napkin-ring sign, and spotty calcification, were more frequently observed in lesions and vessels with FFR ≤ 0.80 (all, *p*-values < 0.05). Among plaque volumes, the percent aggregate plaque volume consistently improved ischemia discrimination independently of stenosis.

**Conclusion:**

CCTA-derived quantification of plaque volumes and identification of high-risk plaque characteristics are associated with ischemia and significantly enhance discrimination of ischemia-causing lesions independently of coronary stenosis severity.

**Key Points:**

***Question*** *Plaque characteristics have been suggested as the missing link between coronary artery stenosis severity and ischemia*.

***Findings*** *High-risk plaque characteristics and larger coronary plaque volumes are associated with ischemia (FFR* *≤* *0.80)*.

***Clinical relevance*** *The addition of CCTA-derived plaque assessment improved the discrimination of ischemia compared with stenosis evaluation alone. Combining coronary stenosis and plaque assessment may improve the non-invasive assessment of patients with coronary artery disease and gatekeeping to the catheterization laboratory*.

**Graphical Abstract:**

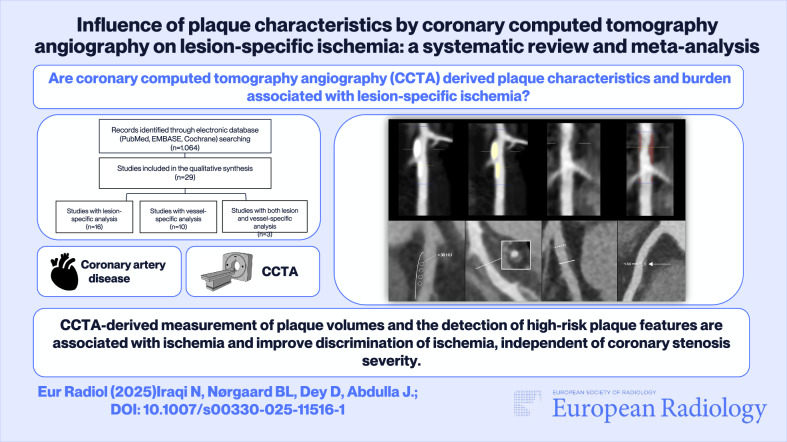

## Introduction

Coronary computed tomography angiography (CCTA) remains an unrivaled non-invasive tool to assess coronary artery disease (CAD) burden and allows detailed assessment of morphological and volumetric plaque characteristics, while invasively measured fractional flow reserve (FFR) is the accepted reference standard for assessing the physiological significance of CAD and guiding decision-making in the catheterization laboratory [1, 2]. The discrepancy between the severity of luminal stenosis assessed by CCTA and its physiological significance is well described [3–6]. It has been demonstrated by CCTA and intravascular imaging that plaque characteristics such as low attenuation plaque (LAP), spotty calcification (SC), and positive remodeling (PR), as well as the atherosclerotic burden, are associated with the presence of physiologically significant CAD [7–9]. Thus, the morphological plaque composition and CAD burden have been proposed as the missing link between stenosis severity and coronary physiology [10]. However, existing studies investigating the correlation between the coronary atherosclerotic phenotype and FFR are of small scale. The aim of this study was to conduct a comprehensive literature review and meta-analysis to further uncover the relationship between CCTA-derived plaque characteristics and invasively measured FFR.

## Methods

### Data sources and search strategy

This systematic literature review was conducted in accordance with the preferred reporting items for systematic review and meta-analysis (PRISMA) guidelines [11]. The study protocol was registered in the PROSPERO database (https://www.crd.york.ac.uk/prospero/, ID: CRD42023468394.) prior to the initiation of the review. PubMed, EMBASE, and The Cochrane Library databases were searched to identify studies investigating the association between CCTA-derived coronary plaque characteristics and invasively measured FFR. The search spanned from January 1st, 2005, to October 1st, 2024, and was restricted to full-text articles published in English. The search strategy included the following keywords used in various relevant combinations: ‘computed tomography angiography’, ‘coronary plaque’, and ‘fractional flow reserve’ along with ‘ischemia’, ‘characteristics’, and ‘features’. Two investigators (N.I. and J.A.) independently conducted the search and reviewed the study titles and abstracts, followed by a full-text review for studies deemed relevant for inclusion. Additionally, a manual review of references to relevant studies was performed to identify any further eligible articles that may have been missed in the initial electronic search. All references were managed in the reference manager program EndNote version 20 (Clarivate Analytics, Philadelphia).

### Study eligibility

We employed the PICO (Population, Intervention, Comparison, Outcome) framework to structure our inclusion criteria and guide the study selection [12]. Studies were eligible for inclusion if they met the following criteria: (P) The studies included patients with suspected or known CAD. (I) Patients underwent CCTA providing quantitative and/or qualitative assessments of per lesion and/or per vessel coronary plaque characteristics. (C) They performed invasive FFR to measure the accurate physiological significance of stenoses, using the generally accepted threshold for decision-making on coronary revascularization ratio of 0.8 to differentiate between the presence (FFR ≤ 0.8) and absence (FFR > 0.8) of ischemia. (O) The primary outcome centered on the association between CCTA-derived plaque characteristics and physiologically significant stenosis, as determined by FFR. Reviews, case reports, and conference abstracts were excluded.

### Data extraction

**Fig. 1:**
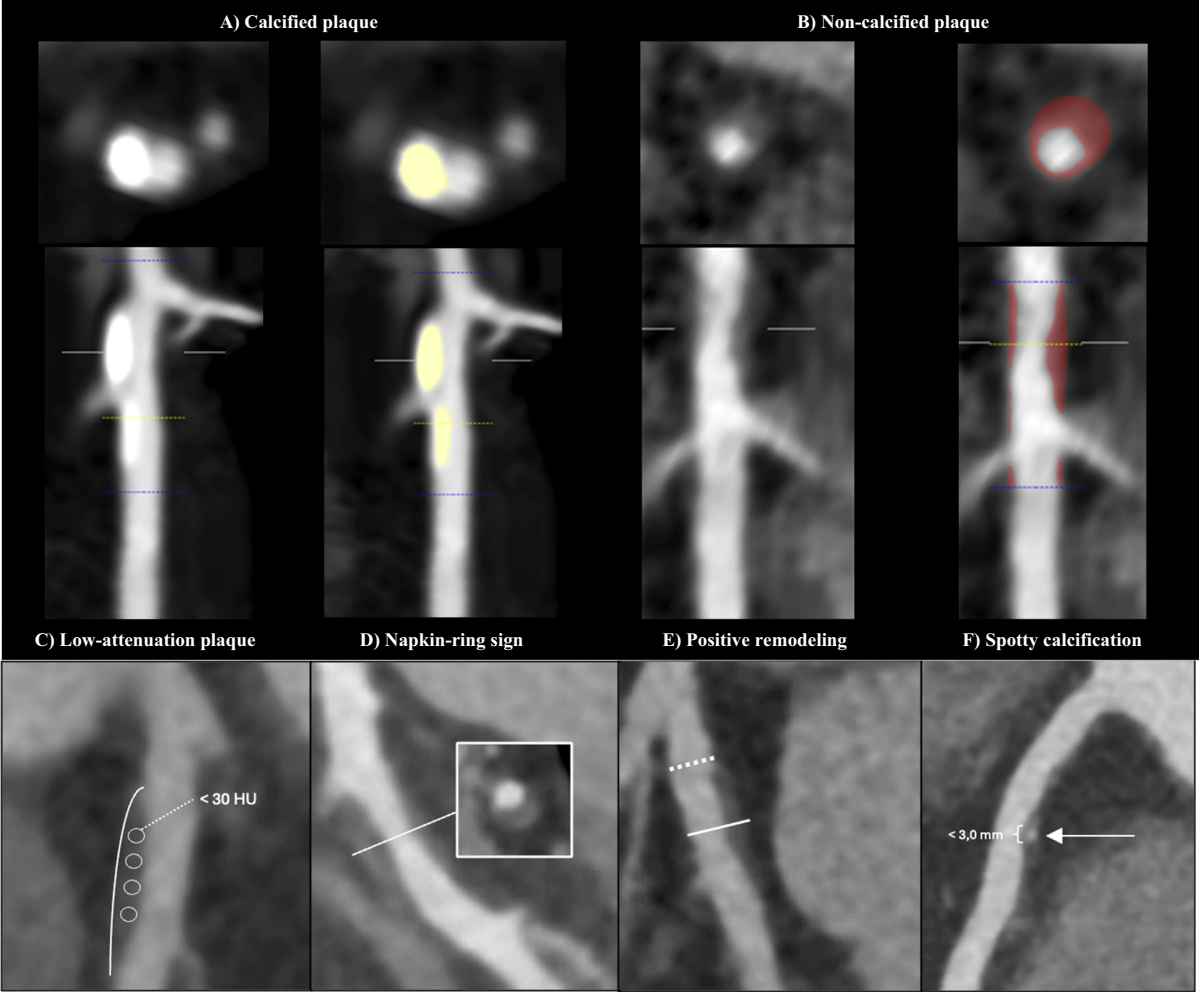
Coronary CT-derived plaque characteristics. Quantification of CP (**A**, yellow) and non-calcified (**B**, red) in cross-sectional and longitudinal view using a semi-automated plaque analysis software (AutoPlaque v.2.9, Cedars-Sinai Medical Center). **C** LAP (plaque with attenuation < 30 HU). **D** NRS (plaque with a core of low CT attenuation surrounded by a rim-like area of higher attenuation). **E** PR (ratio of vessel diameter at plaque site to the diameter of reference segment proximal to the lesion of ≥ 1.1). **F** SC (lesion containing calcium deposit < 3 mm within an arc of < 90° of cross-sectional images)

Study characteristics (sample size, study design, location), type of CT scanner, and plaque analysis tool were extracted from each study along with patient demographic data and key clinical variables reported as means or medians with standard deviation (SD) or ranges. We extracted all available plaque characteristics from studies comparing lesions and/or vessels with abnormal (≤ 0.80) vs normal (> 0.80) FFR. The following CCTA-derived quantitative data were extracted: minimum lumen area (MLA, mm^2^), remodeling index (RI), lesion length (LL, mm), and total plaque volume defined as the absolute volume of all plaque components within the coronary arteries (TPV, mm^3^), aggregate plaque volume (APV) percent (ratio of TPV to the total vessel volume expressed as a percentage, %APV), calcified plaque volume (CPV, mm^3^), non-calcified plaque volume (NCPV, mm^3^), and plaque burden (PB) (ratio of the plaque area to the vessel area expressed as a percentage, %PB). The following qualitative findings were extracted: the presence of high-risk plaque characteristics such as LAP [9], napkin-ring sign (NRS) [13], PR [9], and SC [9] and are illustrated in Fig. 1. Data related to the incremental diagnostic value of plaque characteristics vs luminal stenosis in predicting FFR ≤ 0.80 were extracted when available. Data was extracted independently by two investigators (N.I. and J.A.). Discrepancies were resolved through open consensus.

### Study assessment

Quality assessment of diagnostic accuracy studies (QUADAS-2) score was used for quality assessment of the included studies to evaluate the risk of bias and applicability of primary diagnostic accuracy studies [14]. Quality assessment was conducted independently by two investigators (N.I. and J.A.) and disagreements were resolved through open consensus.

### Statistical analysis

Values of baseline characteristics were pooled either as weighted means or absolute numbers. The weighted mean differences (WMD) method was used for pooled quantitative plaque parameters. Numbers of qualitative plaque characteristics and total populations in each comparison arm were pooled to estimate odds ratios (OR) with a 95% confidence interval (CI). Both analyses were performed on lesion- and vessel-specific levels. Unreported means and SD were calculated from medians and interquartile ranges (IQR) [15]. Heterogeneity was assessed using the traditional Cochrane *Q*-test with *p*-value ≤ 0.1 considered statistically significant and *I*^2^ statistic measured as 0–100%. The *I*^2^ indicated the percentage of variation in the study results attributed to between-study heterogeneity rather than sampling error, and *I*^2^ > 50% was considered significant. Due to the presence of heterogeneity between studies in the majority of analyses, we applied the random effects model when appropriate. For the estimated overall OR and WMD, a *p*-value of 0.05 was considered significant. All analyses and plots were performed using the meta-analysis package of the statistic software program STATA version 16.1 (STATA Corporation).

## Results

### Search results

**Fig. 2:**
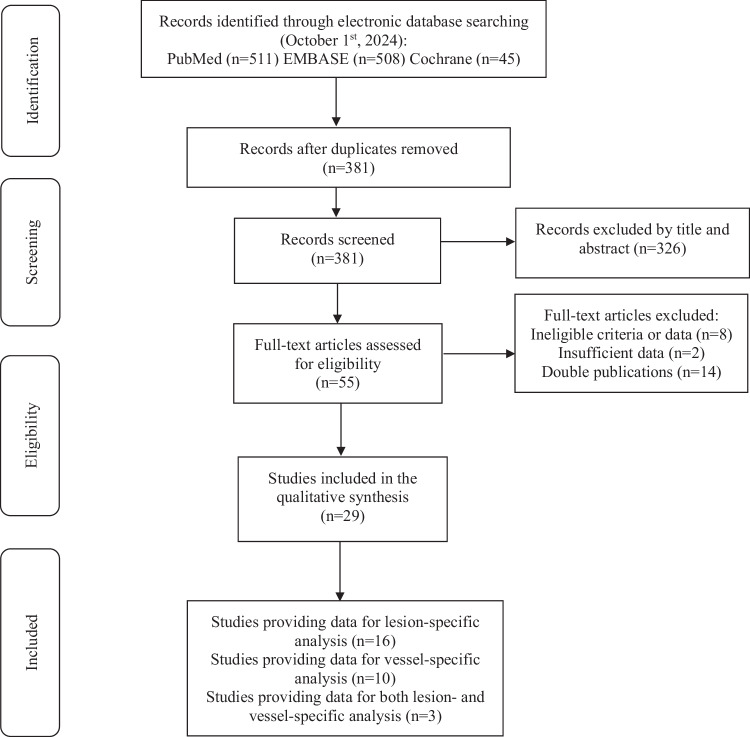
Flow chart of the search process

The search flow is illustrated in Fig. 2. The excluded studies and reasons for exclusion are listed in Table S1. The manual review of study references did not yield any additional studies. A detailed overview of the search process is provided in Table S2.

### Study characteristics

A total of 31 publications were included in this systematic review. Of these, 16 studies reported lesion-specific data [16–31], and 10 studies provided vessel-specific data [32–41]. In four studies, two distinct study populations were analyzed across paired publications: with one publication in each pair reporting lesion-specific data, and the corresponding publication presenting vessel-specific data [42–45]. Additionally, one study included both lesion- and vessel-specific data within the same publication [46]. To prevent duplication, overlapping studies were consolidated, resulting in a total of 29 unique studies, ensuring that each patient was counted only once. All included studies were published between 2010 and 2024. Study characteristics are summarized in Table 1.

**Table 1:** Studies included in the meta-analysis

Study, publication year [ref], *N*	Study design	Location/number of sites	Patients	FFR measurement	CT scanner	Plaque analysis tool	Analysis fashion
Lesion-specific studies							
Kristensen et al [16], *n* = 42	Single-center prospective	Denmark	▪ Indication not specified ▪ Mean age, 61 years ▪ Male gender, 76% ▪ CAG determined intermediate stenosis severity	Invasive FFR	64	^SURE^Plaque, Vitrea 2 v4.0, Vital Image Inc., Toshiba (semi-automated)	Blinded
Nakazato et al [17], *n* = 58	Dual-center prospective	South Korea	▪ Patients with stable angina ▪ Mean age, 63 years ▪ Male gender, 78% ▪ CTA diameter stenosis 30–69%	Invasive FFR	320 and DSCT	AW Advantage, GE Healthcare*	Blinded
Li et al [18], *n* = 61	Single-center retrospective	China	▪ Clinically suspected CAD ▪ Mean age, 65 years ▪ Male gender, 70% ▪ CTA diameter stenosis ≥ 50%	Invasive FFR	128	Syngo; Siemens Medical Solutions^*^	Blinded
Rossi et al [19], *n* = 99	Dual-center retrospective	The Netherlands, UK	▪ Patients with stable angina ▪ Mean age, 61 years ▪ Male gender, 77% ▪ CTA diameter stenosis < 50 to > 70%	Invasive FFR	64 and 128	QAngio CT RE v1.3.61, Medis (semi-automated)	Not specified
FIGURE-OUT Doh et al [20], *n* = 151	Multi-center prospective	South Korea/3	▪ Indication not specified ▪ Mean age, 63 years ▪ Male gender, 72% ▪ CTA diameter stenosis 30–70%	Invasive FFR	64	AW Advantage 4.5, GE Healthcare^*^	Blinded
Opolski et al 2014 [21], *n* = 61	Single-center prospective	Poland	▪ Indication not specified ▪ Mean age, 63 years ▪ Male gender, 64% ▪ CTA diameter ≥ 50–80%	Invasive FFR	DSCT	^SURE^Plaque, Vitrea v6.3, Vital Image Inc., Toshiba (semi-automated)	Blinded
DeFACTO Park/Rizvi et al [42, 43], *n* = 252	Multi-center prospective	Belgium, Canada, Latvia, South Korea, US/17	▪ Clinically suspected CAD ▪ Mean age, 63 years ▪ Male gender, 71% ▪ CTA diameter stenosis < 25 to > 70%	Invasive FFR	≥ 64	QAngio CT RE v2.02, Medis (semi-automated)	Blinded
Hell et al [22], *n* = 59	Single-center retrospective	Germany	▪ Indication not specified ▪ Mean age, 64 years ▪ Male gender, 75% ▪ CTA diameter stenosis < 50% to > 75%	Invasive FFR	128	AutoPlaq v9.5, Cedars-Sinai Medical Center (semi-automated)	Blinded
Wang et al [23], *n* = 49	Single-center retrospective	US	▪ Clinically suspected or known CAD ▪ Mean age, 62 years ▪ Male gender, 67% ▪ CTA diameter stenosis < 50 to > 70%	Invasive FFR	DSCT	Not specified	Blinded
NXT Gaur et al [24], *n* = 254	Post hoc sub-study multi-center prospective	Japan, Australia, England, Germany, South Korea, Scotland, Latvia, Denmark/10	▪ Clinically suspected stable CAD ▪ Mean age, 64 years ▪ Male gender, 64% ▪ CTA diameter stenosis < 25–100%	Invasive FFR and FFR_CT_	≥ 64	AutoPlaque v9.7, Cedars-Sinai Medical Center (semi-automated)	Blinded
Zhang et al [25], *n* = 49	Dual-center retrospective	Singapore, China	▪ Clinically suspected or known CAD ▪ Mean age, 59 years ▪ Male gender, 61% ▪ CTA diameter stenosis < 25–100%	Invasive FFR and FFR_CT_	≥ 64	QAngio CT RE v3.0, Medis (semi-automated)	Blinded
Yu et al [26], *n* = 180	Single-center retrospective	China	▪ Clinically suspected CAD ▪ Mean age, 63 years ▪ Male gender, 64% ▪ Diameter stenosis not specified	Invasive FFR and FFR_CT_	128	Coronary Plaque Analysis v2.0, Syngo.via Frontier, Siemens (semi-automated)	Blinded
Doeberitz et al 2019 [27], *n* = 84	Single-center retrospective	US	▪ Clinically suspected or known CAD ▪ Mean age, 61 years ▪ Male gender, 65% ▪ CTA diameter stenosis < 25–100%	Invasive FFR and FFR_CT_	DSCT	Coronary Plaque Analysis v4.2.0, Syngo.via Frontier, Siemens (semi-automated)	Blinded
Du et al [28], *n* = 45	Single-center retrospective	China	▪ Clinically suspected or known CAD ▪ Mean age, 60 years ▪ Male gender, 76% ▪ CAG diameter stenosis 30–90%	Invasive FFR	128	Not specified	Not specified
Li et al [29], *n* = 149	Single-center retrospective	China	▪ Clinically suspected or known CAD ▪ Mean age, 62 years ▪ Male gender, 64% ▪ CTA diameter stenosis 30–90%	Invasive FFR	DSCT	Coronary plaque analysis v2.0, Siemens (semi-automated)	Blinded
3V FFR-FRIENDS Yang et al [45], *n* = 643	Multi-center prospective and retrospective	China, Japan, South Korea, Singapore, Taiwan/12	▪ Clinically suspected CAD ▪ Mean age, 66 years ▪ Male gender, 75% ▪ Diameter stenosis not specified	Invasive FFR	Not specified	QAngio CT RE v2.1.9.1, Medis (semi-automated)	Blinded
Lee et al [30], *n* = 159	Multi-center prospective	South Korea/4	▪ Clinically suspected CAD ▪ Mean age, 63 years ▪ Male gender, 74% ▪ CAG diameter stenosis 40–70%	Invasive FFR	≥ 64	QAngio CT RE, Medis (semi-automated)	Blinded
Long et al [31], *n* = 138	Single-center retrospective	China	▪ Clinically suspected CAD ▪ Mean age, 64 years ▪ Male gender, 67% ▪ Diameter stenosis not specified	Invasive FFR	320	CoronaryDoc, Shukun Technology (automated)	Not specified
Vessel-specific studies							
DeFACTO Park/Rizvi et al [42, 43], *n* = 252	Multi-center prospective	Belgium, Canada, Latvia, South Korea, US/17	▪ Clinically suspected CAD ▪ Mean age, 63 years ▪ Male gender, 71% ▪ CTA diameter stenosis < 25 to > 70%	Invasive FFR	≥ 64	QAngio CT RE v2.02, Medis (semi-automated)	Blinded
PACIFIC Driessen et al [32], *n* = 179	Post hoc substudy Single-center prospective	The Netherlands	▪ Clinically suspected CAD ▪ Mean age, 58 years ▪ Male gender, 64% ▪ CTA diameter stenosis 0% to > 70%	Invasive FFR	256	Comprehensive Cardiac Analysis, Philips Healthcare (semi-automated)	Blinded
3V FFR-FRIENDS Lee et al 2019 [44], *n* = 299	Substudy Multi-center prospective	China, Japan, South Korea, Singapore, Taiwan/12	▪ Clinically suspected CAD ▪ Mean age, 63 years ▪ Male gender, 77% ▪ CTA diameter stenosis > 30%	Invasive FFR	≥ 64	Intellispace Portal, Phillips Healthcare (semi-automated)	Blinded
Kawai et al [33], *n* = 297	Dual-center retrospective	Japan	▪ Clinically suspected or known CAD ▪ Mean age, 69 years ▪ Male gender, 77% ▪ CTA diameter stenosis 50–90%	Invasive FFR	320	QAngio CT RE v2.02, Medis (semi-automated)	Blinded
Yin et al [34], *n* = 132	Single-center retrospective	China	▪ Indication not specified ▪ Mean age, 61 years ▪ Male gender, 66% ▪ CTA diameter stenosis 30–85%	Invasive FFR	DSCT	Coronary Plaque Analysis v2.0.3, Syngo.via Frontier, Siemens (semi-automated)	Blinded
CT-FFR CHINA Zhao et al [35], *n* = 317	Multi-center prospective	China/5	▪ Clinically suspected CAD ▪ Mean age, 59 years ▪ Male gender, 68% ▪ CTA diameter stenosis 30–90%	Invasive FFR and FFR_CT_	≥ 64	Syngo.via, Frontier, Siemens (semi-automated)	Blinded
Ma et al [36], *n* = 227	Multi-center retrospective	China/7	▪ Clinically suspected CAD ▪ Mean age, 62 years ▪ Male gender, 67% ▪ CAG diameter stenosis 30–90%	Invasive FFR	64	Coronary Plaque Analysis v1.2.0, Syngo.via, Frontier, Siemens (semi-automated)	Blinded
Velangi et al [37], *n* = 134	Single-center retrospective	US	▪ Clinically suspected CAD ▪ Mean age, 62 years ▪ Male gender, 62% ▪ CTA diameter stenosis < 50% to > 70%	Invasive FFR	128	Aquarius platform, TeraRecon (semi-automated)	Blinded
Tang et al [38], *n* = 144	Single-center retrospective	China	▪ Known CAD ▪ Mean age, 62 years ▪ Male gender, 67% ▪ Diameter stenosis not specified	Invasive FFR and FFR_CT_	320	Vitrea FX v4.0, Vital Images, Canon (automated)	Blinded
Yan et al [39], *n* = 144	Single-center retrospective	China	▪ Clinically suspected or known stable CAD ▪ Mean age, 57 years ▪ Male gender, 77% ▪ CTA diameter stenosis 30–90%	Invasive FFR and FFR_CT_	DSCT	QAngio CT RE v3.0, Medis (semi-automated)	Blinded
Lee et al [40], *n* = 127	Multi-center retrospective	South Korea, Japan, Australia, Spain	▪ Clinically suspected stable CAD or ACS ▪ Mean age, 65 years ▪ Male gender, 74% ▪ Diameter stenosis not specified	Invasive FFR and iFR	≥ 64	Intellispace Portal, Phillips Healthcare (semi-automated)	Blinded
Wang et al [41], *n* = 133	Dual-center prospective	Singapore	▪ Clinically suspected or known CAD ▪ Mean age, 63 years ▪ Male gender, 74% ▪ CTA diameter stenosis 30–90%	Invasive FFR and FFR_CT_	256, 320, and 640	QAngio CT RE v3.2, Medis (semi-automated)	Blinded
Lesion-and vessel-specific study							
Kato et al [46], *n* = 49	Single-center retrospective	Japan	▪ Indication not specified ▪ Mean age (FFR ≤ 0.8), 68 years ▪ Mean age (FFR > 0.8), 70 years ▪ Male gender, 82% ▪ QCA diameter stenosis 30–69%	Invasive FFR	320	QAngio CT RE v2.1 RC4, Medis (semi-automated)	Blinded

*ACS* acute coronary syndrome, *CAD* coronary artery disease, *CAG* coronary angiography, *CT* computed tomography, *CTA* computed tomography angiography, *DL* deep-learning, *DSCT* dual-source CT scanner, *FFR* fractional flow reserve, *FFR*_*CT*_ computed tomography derived fractional flow reserve, *iFR* instantaneous wave-free ratio, *MDCT* multidetector computed tomography, *RE* research edition, *UK* United Kingdom, *US* United States, *QCA* quantitative coronary angiography

^*^ Not specified if manual, semi-automated, or automated plaque analysis software

### Patient characteristics

A total of 4416 patients with a mean age of 63 ± 9 years were included in the meta-analysis, of whom 71% were male. Symptoms were reported in 13 studies [17–21, 24, 30–32, 37, 40, 43, 45], with the majority of the patients presenting with stable angina. Patient characteristics are shown in Table 2.

**Table 2:** Patient baseline characteristics

Variable	Studies providing data	Values
Patients, *n*	29	4416
Age (years, SD)	29	62.9 ± 9.3
Male, *n* (%)	29	3119/4416 (71)
Hypertension, *n* (%)	26	2423/3476 (64)
Diabetes mellitus, *n* (%)	26	1097/3477 (32)
Dyslipidemia, *n* (%)	26	2033/3477 (59)
History of smoking, *n* (%)	26	1106/3326 (32)
Symptoms, *n* (%)	13	
Stable angina	13	1546 /2283 (68)
Atypical angina	6	297/974 (30)
Non-cardiac angina	3	64/652 (10)
Dyspnea	3	39/582 (7)
Silent ischemia	3	23/212 (11)
Acute coronary syndrome	6	243/1141 (21)
Asymptomatic	2	71/384 (18)
Number of analyzed lesions/patients	19	3923/2792
Number of lesions with low/normal FFR, *n* (%)	19	1363/2560 (35/65)
Number of analyzed vessels/patients	12	3520/2301
Number of vessels with low/normal FFR, *n* (%)	12	1326/2194 (38/62)

Values are presented as means (± standard deviation, SD) and numbers (percentages) if not stated otherwise

*FFR* fractional flow reserve

### Imaging protocols and plaque assessment

All studies utilized contrast-enhanced scan protocols for coronary plaque analysis. Semi-automated postprocessing plaque analysis software was employed in 22 studies (78%) [16, 19, 21, 22, 24–27, 29, 30, 32–37, 39–46], while fully automated software was used in two studies (6%) [31, 38]. Three studies did not report details on assessment automation [17, 18, 20], and the remaining two studies did not provide any analysis software information [23, 28] (Table 1). In studies reporting NCPV and CPV, scan-specific attenuation thresholds were applied in two [22, 24], whereas the remaining studies applied different fixed attenuation thresholds as detailed in Table 3. Similarly, definitions of qualitative plaque characteristics varied across studies (Table 3).

**Table 3:** Definition of quantitative and qualitative plaque characteristics

Plaque characteristics	Definitions [ref]
RI	▪ Ratio of lesion vessel area to the vessel area of reference proximal area [16, 18, 23, 24, 27, 32, 38]. ▪ Ratio of maximal lesion vessel diameter of the lesion to the proximal reference vessel diameter [26, 30, 33, 35–37, 40, 42, 43] ▪ Ratio of a maximal cross-section of the vessel to the cross-section of the medial healthy reference vessel [34]
TPV	▪ Sum of all contiguous voxels between the outer vessel contour and the lumen border [16, 21]. ▪ Difference between vessel volume and lumen volume [25]. ▪ Sum of the volumes of separate plaques along each coronary artery [32, 37]
NCPV	▪ Volume with HU value 17–124 [27] ▪ Volume with HU value < 190 [29] ▪ Volume with HU value < 150 [32] ▪ Volume with HU value 30–190 [34] ▪ Any discernible structure that could be assigned to the coronary artery wall, had a CT number below the contrast-enhanced coronary lumen but above the surrounding connective tissue, and could be identified in at least two independent planes [36] ▪ Volume with HU value −30 to 350 [38, 45]
CPV	▪ Volume with HU value 350–1000 [21] ▪ Volume with HU value > 511 [27] ▪ Volume with HU value ≥ 150 [32] ▪ Volume with HU value 350–1300 [34] ▪ Volume with HU value > 350 [38, 45] ▪ Voxels with HU value > 130 [39]
LD-NCPV/LAPV	▪ Volume with HU value < 30 [24, 25, 38–40]
APV%	▪ Ratio of APV to the vessel volume, reported as a percentage [17, 23, 24, 33, 39, 42–44]
PB%	▪ Difference between vessel area and lumen area divided by vessel area, reported as a percentage [16, 19, 21, 25] ▪ Ratio of plaque cross-sectional area to the vessel cross-sectional area, reported as a percentage [18, 26, 27, 32] ▪ Ratio of plaque volume to vessel volume, reported as a percentage [34, 35, 38, 46]
LAP	▪ Presence of any voxel < 30 HU within a coronary plaque [26, 32, 33, 35–37, 42, 43, 45, 46] ▪ Plaque with average density ≤ 30 HU from three random region-of-interest measurements with approximately 0.5–1.0 mm^2^ in non-calcified portion of plaque [30, 40, 44]
NRS	▪ A rim-link area of higher attenuation surrounding a lower CT attenuation within an atherosclerotic plaque [25–27, 30, 32, 35, 36, 38, 40, 44, 45] ▪ A ring-like high-attenuation zone surrounding a coronary atherosclerotic plaque and the CT attenuation value of the ring was greater than that of the plaque but ≤ 130 HU to exclude calcium deposition [34]
SC	▪ An intralesion calcific plaque < 3 mm in length that comprised < 90° of the lesion circumference [24, 26, 27, 32, 35, 42, 43, 46] ▪ Average density > 130 HU, diameter < 3 mm in any direction [45] ▪ Average density > 130 HU, diameter < 3 mm in any direction, length of the calcium < 1.5× the vessel diameter, and width of the calcification < 2/3 of the vessel diameter [30, 36, 40, 44] ▪ CP with a maximum diameter of < 3 mm in any direction [37, 38]
PR	▪ RI ≥ 1.1 [26, 30, 33, 36, 37, 40, 42–46] ▪ RI > 1.1 [24, 32, 35]

*APV* aggregate plaque volume (summation of all contiguous plaque areas from the coronary artery ostium to the distal portion of the lesion), *CPV* calcified plaque volume (mm^3^), *LAP* low attenuation plaque, *LD-NCP* low-density non-calcified plaque volume (mm^3^), *LL* lesion length (mm), *MLA* minimum lumen area (mm^2^), *NCPV* non-calcified plaque volume (mm^3^), *NRS* napkin-ring sign, *PR* positive remodeling, *RI* remodeling index, *SC* spotty calcification, *TPV* total plaque volume (mm^3^)

### Meta-analysis of the association between quantitative and qualitative coronary plaque characteristics and lesion-specific ischemia

**Fig. 3:**
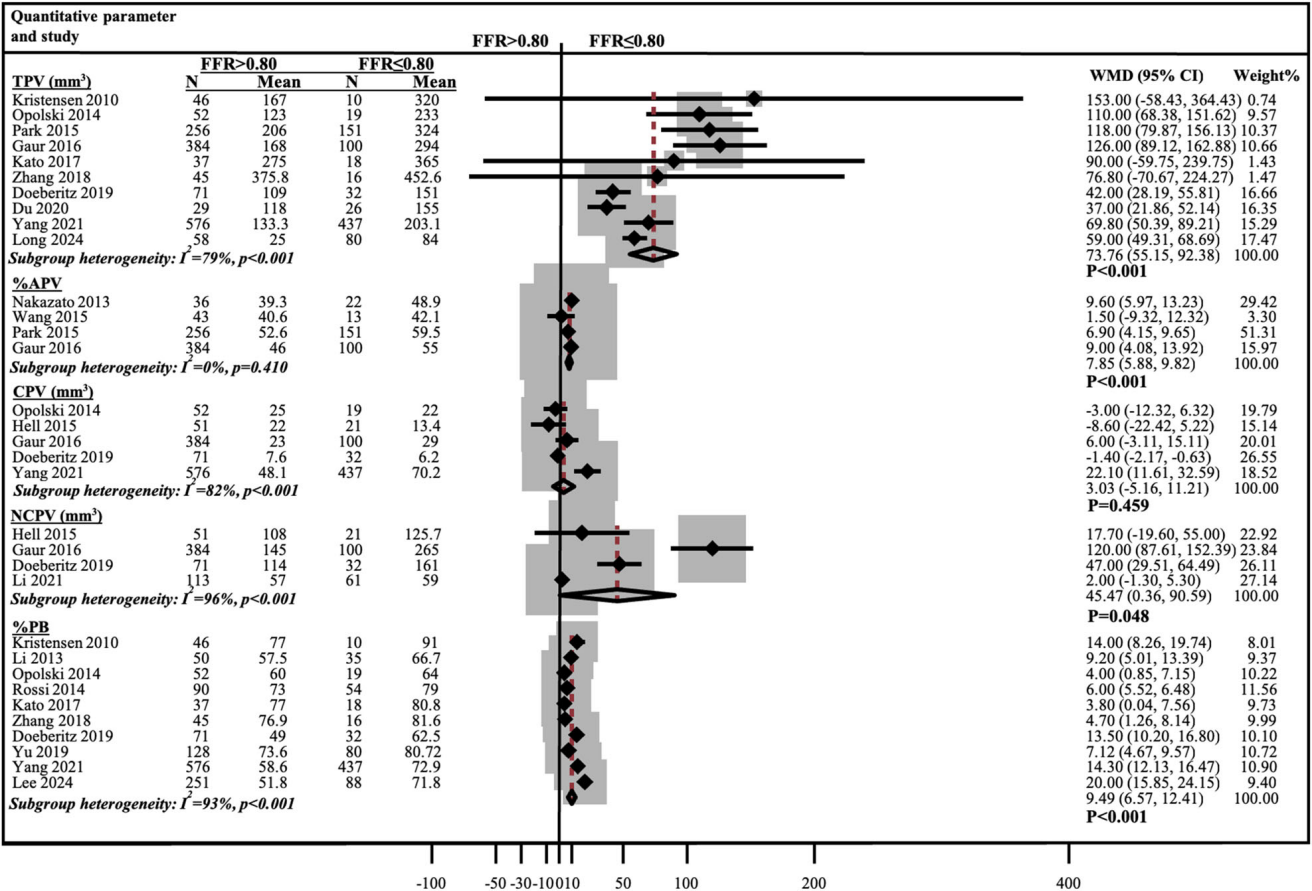
Meta-analysis of quantitative plaque characteristics comparing lesions with FFR ≤ 0.80 vs > 0.80. APV, aggregate plaque volume (%); CI, confidence interval; CPV, calcified plaque volume (mm^3^); FFR, fractional flow reserve; N, number; NCPV, non-calcified plaque volume (mm^3^); TPV, total plaque volume (mm^3^); PB, plaque burden (%); WMD, weighted mean difference

**Fig. 4:**
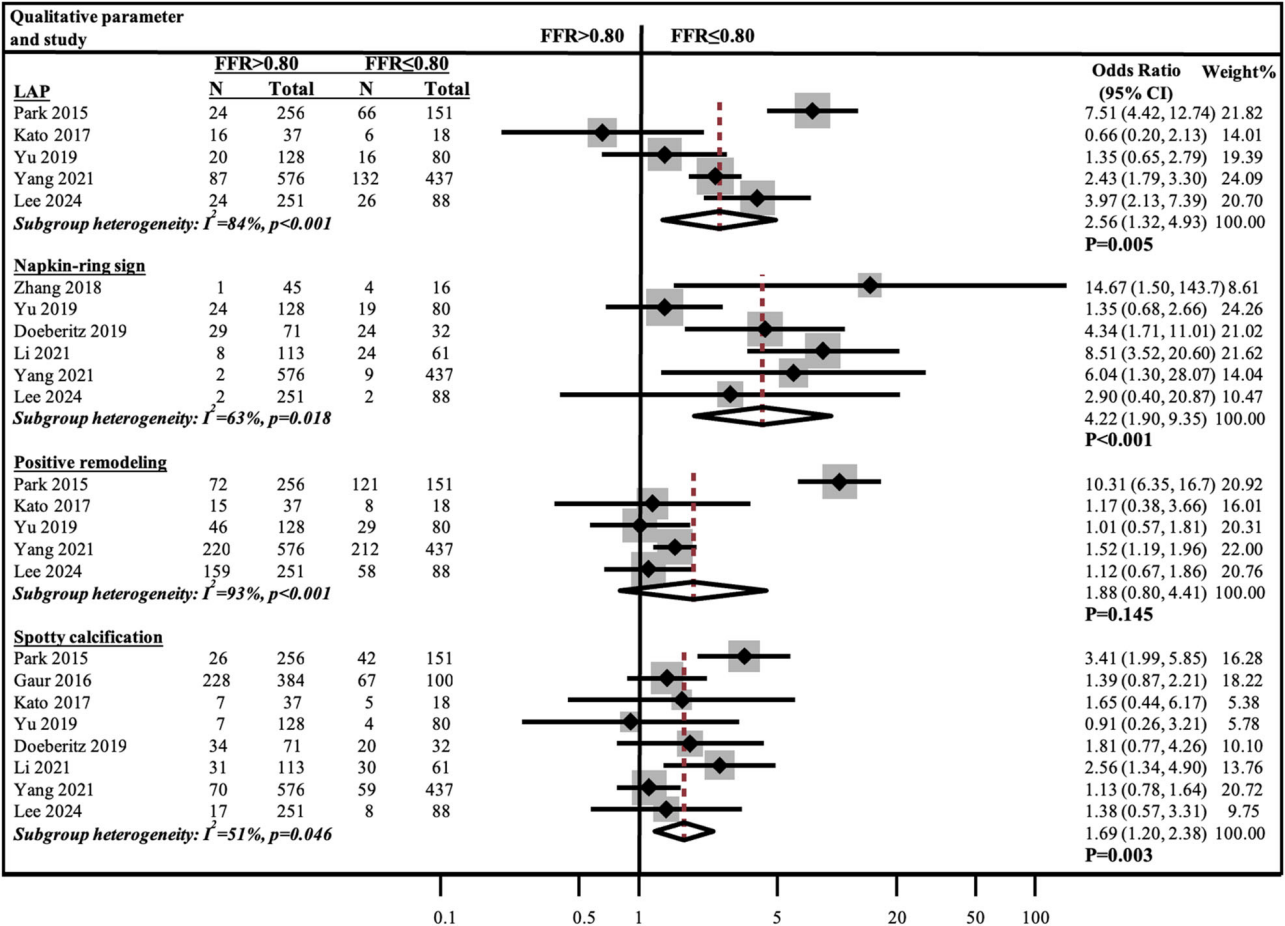
Meta-analysis of qualitative plaque characteristics comparing lesions with FFR ≤ 0.80 vs > 0.80. CI, confidence interval; FFR, fractional flow reserve; LAP, low attenuation plaque; N, number

Pooled data from 2792 patients including 1363 (35%) lesions with FFR ≤ 0.80 and 2560 (65%) lesions with FFR > 0.80 were analyzed. Lesions with FFR ≤ 0.80 demonstrated significantly smaller MLA, higher RI, and longer LL, all *p* ≤ 0.032 (Fig. S1). TPV, %APV, NCPV, and %PB were significantly greater in lesions with FFR ≤ 0.80, all *p* < 0.05 (Fig. 3). Conversely, CPV showed no significant WMD between the two groups of 3 mm^3^ (*p* = 0.459). LAP, NRS, and SC were more frequently observed in lesions with FFR ≤ 0.80 compared to FFR > 0.8 (OR 2.56, 95% CI: 1.32–4.93, *p* = 0.005; OR 4.22, 95% CI: 1.90–9.35, *p* < 0.001; OR 1.69, 95% CI: 1.20–2.38, *p* = 0.003, respectively). PR was not significantly associated with FFR ≤ 0.80 (Fig. 4). Subgroup heterogeneity ranged from moderate to high (*I*^2^ = 51–96%, all *p* < 0.05).

### Meta-analysis of the association between quantitative and qualitative coronary plaque characteristics and vessel-specific ischemia

**Fig. 5:**
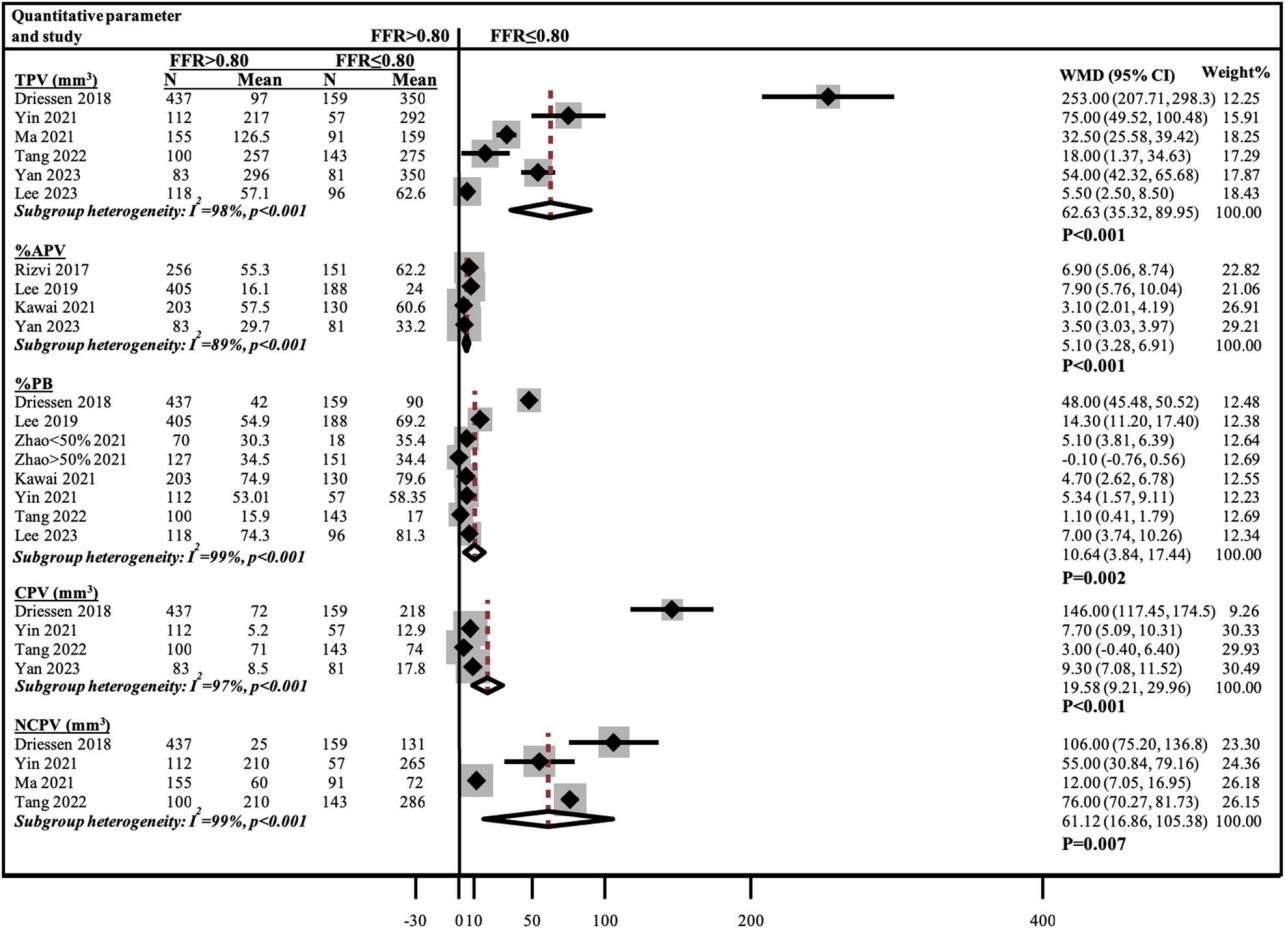
Meta-analysis of quantitative plaque characteristics comparing vessels with FFR ≤ 0.80 vs > 0.80. APV, aggregate plaque volume (%); CI, confidence interval; CPV, calcified plaque volume (mm^3^); FFR, fractional flow reserve; N, number; NCPV, non-calcified plaque volume (mm^3^); TPV, total plaque volume (mm^3^); PB, plaque burden (%); WMD, weighted mean difference

**Fig. 6:**
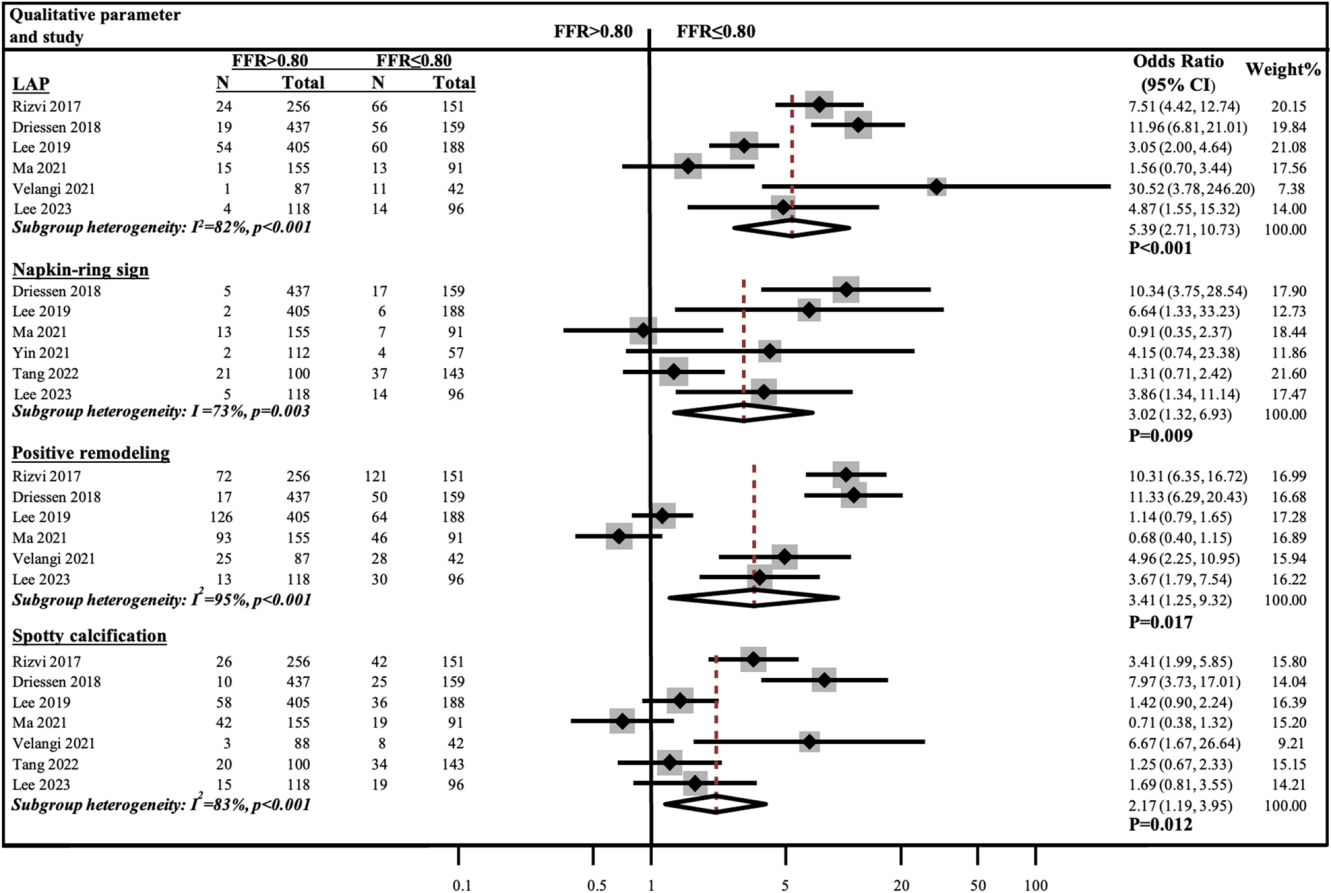
Meta-analysis of qualitative plaque characteristics comparing vessels with FFR ≤ 0.80 vs > 0.80. CI, confidence interval; FFR, fractional flow reserve; LAP, low attenuation plaque; N, number

Pooled analyses on 2301 patients included 1326 (38%) coronary vessels with FFR ≤ 0.80 and 2194 (62%) vessels with FFR > 0.80. MLA was directly associated with FFR, while LL, was inversely related to FFR. Four studies [34, 38, 40, 44] compared RI and showed no significant WMD of −0.01 (95% CI: −0.05 to 0.04), *p* = 0.716 (Fig. S2). Vessels with FFR ≤ 0.80 demonstrated significantly greater values of TPV, %APV, NCPV, CPV, and %PB compared to vessels FFR > 0.80, all *p* ≤ 0.007 (Fig. 5). LAP, NRS, PR, and SC were more prevalent in vessels with FFR ≤ 0.80 compared to FFR > 0.80 (OR 5.39, 95% CI: 2.71–10.73, *p* < 0.001; OR 3.01, 95% CI: 1.32–6.91, *p* = 0.009; OR 3.41, 95% CI: 1.25–9.32, *p* = 0.017; OR 2.17, 95% CI: 1.19–3.95, *p* = 0.012, respectively) (Fig. 6). Heterogeneity across subgroups was high (*I*^2^ = 73–99%, *p* ≤ 0.003) except for RI, which showed borderline heterogeneity (*I*^2^ = 49%, *p* = 0.11).

### Data combining luminal stenosis and plaque characteristics for prediction of FFR ≤ 0.80

Ten studies [17–21, 24–27, 42] provided data on the discriminatory performance of luminal stenosis severity or models that integrate plaque characteristics for predicting FFR ≤ 0.80. Four studies [32, 34, 37, 39] reported data on the vessel-specific discriminatory performance. The discriminatory performance varied across the included studies (Table 4). In three studies [17, 39, 42], %APV consistently enhanced the AUC. Improvement in discriminatory performance was evident in models that incorporated coronary plaque volumes alongside diameter stenosis [17, 24, 32, 39, 42]. The most substantial discriminatory improvement was observed in models [25, 27, 32, 37, 42] that combined both qualitative and quantitative plaque characteristics in addition to luminal stenosis.

**Table 4:** Receiver operator curve produced area under curve (AUC) models for discrimination of ischemia

Study author and publication year [ref]	Model	AUC1 (95% CI)	AUC2 (95% CI)
Lesion-specific studies			
Nakazato et al [17]	DS vs %APV DS vs MLA DS vs DS + %APV DS vs MLA + %APV	0.68 (0.54–0.83) 0.68 (0.54–0.83) 0.68 (0.54–0.83) 0.68 (0.54–0.83)	0.85 (0.74–0.97) 0.78 (0.64–0.91) 0.88 (0.78–0.99) 0.89 (0.79–0.99)
Li et al [18]	DS vs LL DS vs MLA DS vs %PB	0.76 (0.66–0.86) 0.76 (0.66–0.86) 0.76 (0.66–0.86)	0.77 (0.67–0.87) 0.76 (0.66–0.86) 0.74 (0.63–0.84)
Rossi et al [19]	DS vs LL DS vs MLA DS vs %PB	0.82 (0.75–0.89) 0.82 (0.75–0.89) 0.82 (0.75–0.89)	0.66 (0.57–0.75) 0.82 (0.75–0.89) 0.80 (0.73–0.87)
Doh et al [20]	DS vs MLA	0.66 (0.58–0.73)	0.71 (0.64–0.78)
Opolski et al [21]	AS (> 69%) vs MLA (≤ 3 mm^2^) AS (> 69%) vs LL (> 18.5 mm) AS (> 69%) vs TPV (> 150 mm^3^)	0.67 (0.55–0.78) 0.67 (0.55–0.78) 0.67 (0.55–0.78)	0.77 (0.66–0.86) 0.86 (0.76–0.93) 0.86 (0.75–0.93)
Park et al [42]	AS vs AS + %APV AS vs AS + %APV + APCs	0.72 0.72	0.79 0.86
Gaur et al [24]	DS (> 50%) vs LD-NCPV (≥ 30 mm^3^) DS (> 50%) vs DS (> 50%) + LD-NCPV (≥ 30 mm^3^)	0.71 (0.67–0.76) 0.71 (0.67–0.76)	0.73 (0.67–0.78) 0.79 (0.74–0.84)
Zhang et al [25]	DS (≥ 50%) vs DS (≥ 50%) + NPV + NRS	0.70 (0.59–0.82)	0.81 (0.68–0.93)
Yu et al [26]	DS (> 70.5%) vs LL (> 10.6 mm) DS (> 70.5%) vs MLA (≤ 1.95 mm^2^) DS (> 70.5%) vs %PB (> 77.9)	0.75 (0.69–0.81) 0.75 (0.69–0.81) 0.75 (0.69–0.81)	0.65 (0.58–0.72) 0.73 (0.66–0.79) 0.74 (0.67–0.80)
Doeberitz et al [27]	DS (≥ 50%) vs LL DS (≥ 50%) vs RI DS (≥ 50%) vs NCPV DS (≥ 50%) vs NRS DS (≥ 50%) vs DS (≥ 50%) + LL + RI + NCPV + NRS	0.61 (0.51–0.70) 0.61 (0.51–0.70) 0.61 (0.51–0.70) 0.61 (0.51–0.70) 0.61 (0.51–0.70)	0.70 0.62 0.62 0.67 0.83 (0.75–0.90)
Vessel-specific studies			
Driessen et al [32]	DS vs DS + NCPV DS vs DS + NCPV + PR	0.86 0.86	0.89 0.90
Yin et al [34]	MDS vs %PB MDS vs CPV MDS vs TPV	0.77 0.77 0.77	0.83 0.60 0.59
Velangi et al [37]	DS vs PR DS vs LL (per 1 mm) DS vs SC DS vs LAP DS vs LAP + LL (per 1 mm) DS vs LD-NCPV DS vs NCPV DS vs CPV DS vs TPV	0.53 (0.47–0.59) 0.53 (0.47–0.59) 0.53 (0.47–0.59) 0.53 (0.47–0.59) 0.53 (0.47–0.59) 0.53 (0.47–0.59) 0.53 (0.47–0.59) 0.53 (0.47–0.59) 0.53 (0.47–0.59)	0.69 0.83 (0.75–0.91) 0.58 0.64 (0.55–0.73) 0.87 (0.80–0.94) 0.53 0.54 0.59 0.50
Yan et al [39]	DS (≥ 50%) vs DS (≥ 50%) + LD-NCPV (≥ 76.23 mm^3^) DS (≥ 50%) vs DS (≥ 50%) + %APV (≥ 28.9%) DS (≥ 50%) vs DS (≥ 50%) + LD-NCPV (≥ 76.23 mm^3^) + %APV (≥ 28.9%)	0.65 (0.57–0.72) 0.65 (0.57–0.72) 0.65 (0.57–0.72)	0.70 (0.63–0.77) 0.72 (0.64–0.79) 0.74 (0.67–0.81)
Lesion- and vessel-specific studies			
Kato et al [46]	DS vs Lesion %PV DS vs Vessel %PV	0.50 (0.34–0.67) 0.50 (0.34–0.67)	0.65 (0.48–0.79) 0.76 (0.61–0.87)

The AUC models for discrimination of ischemia by invasive FFR (≤ 0.80), comparing stenosis severity quantification alone (AUC1) and after adding plaque characteristics (AUC2)

*APCs* atherosclerotic plaque characteristics (PR, LAP, and SC), *APV* aggregate plaque volume (%), *AS* area stenosis (%), *AUC* area under the curve, *CPV* calcified plaque volume (mm^3^), *DS* diameter stenosis (%), *LAP* low attenuation plaque, *LL* lesion length (mm), *LD-NCPV* low-density non-calcified plaque volume (mm^3^), *MDS* maximum diameter stenosis (mm), *MLA* minimal lumen area (mm^2^), *NCPV* non-calcified plaque volume (mm^3^), *NPV* normalized plaque volume (calculated as ratio of plaque volume to representative lesion volume (LL × MLA), *NRS* napkin-ring sign, *PR* positive remodeling, *RI* remodeling index, *SC* spotty calcification, *TPV* total plaque volume (mm^3^)

### Study quality assessment

Three studies [25, 26, 38] had a high risk of bias in patient selection, whereas four studies [19, 21, 29, 33] had an unclear risk of bias, and the remaining studies had a low risk of bias (Table S3). Ten of the included 29 studies were prospectively designed [16, 17, 20, 21, 24, 30, 32, 35, 41–43], while 18 were based on retrospective data [18, 19, 22, 23, 25–29, 31, 33, 34, 36–40, 46], and one study combined prospective enrollment with a retrospective registry [45]. Reason for referral was not provided in 7 studies [16, 17, 20–22, 34, 46] and stenosis severity was not specified in five studies [26, 31, 38, 40, 45]. All included studies reported that patients underwent both CCTA followed by ICA and FFR as a part of the evaluation process. For the index test domain, the majority of studies exhibited a low risk of bias. In most studies, both CCTA and invasive FFR readers reported data independently, in a blinded fashion. The temporal alignment between the CCTA and ICA/FFR was not specified in five studies [19, 22, 32, 34, 44]. One study [25] accepted time intervals of up to six months, whereas the remaining studies reported intervals ranging from one to 90 days. In the reference standard domain, 27 studies used an FFR threshold ≤ 0.80 to define ischemia, while two studies applied an alternative threshold of ≤ 0.75 [16, 31]. Applicability across all QAUDAS-2 domains was deemed high in relevance to the research question, with the exception of one study [44] that exclusively included patients with three-vessel disease representing a highly selected population. The detailed QUADAS-2 scores for all included studies are provided in Table S3.

## Discussion

This comprehensive meta-analysis of a large number of studies elucidates the intricate and important relationship between coronary plaque characteristics and FFR. The findings demonstrate that quantitative plaque characteristics such as MLA were significantly associated with FFR, while LL, TPV, %APV, NCPV, and %PB demonstrated an inverse association with FFR. Qualitative plaque characteristics, including LAP, NRS, and SC, were more frequently observed in lesions and vessels with FFR ≤ 0.80. Moreover, integrating CCTA-derived quantitative and qualitative high-risk plaque characteristics improved the discrimination of ischemia beyond luminal stenosis evaluation alone.

This study demonstrated that smaller MLA and longer LL are associated with FFR ≤ 0.80. As MLA is a measure of the residual lumen, smaller values correlate with reduced coronary flow and a higher likelihood of ischemia, aligning with prior CTA and intravascular ultrasound (IVUS) studies that underscore MLA’s critical role in determining the hemodynamic significance of coronary lesions [47, 48]. These findings are of no surprise since pressure is inversely proportional to the radius in the fourth power, thus a relatively small change in radius may lead to a significant translesional pressure drop. Similarly, longer LL associated with low FFR corroborates earlier evidence suggesting that extensive plaque accumulation imposes a greater physiological burden, contributing to higher pressure gradients and reduced FFR values [49]. In this study RI was significantly associated with ischemia on a per-lesion but not on a per-vessel basis. The latter finding is partially in contrast to previous studies showing that the presence of an RI is associated with unfavorable cardiovascular outcomes (9). Notably, relatively few patients were available in the RI per-vessel analysis, thus potentially lacking statistical power.

Consistently, across both per-lesion and per-vessel analyses, higher TP and NCP volumes, along with greater %APV and %PB, were significantly associated with ischemia. These findings underscore that plaque composition and overall burden are important determinants of the functional significance of CAD. Similar associations have been demonstrated in earlier CTA studies, where larger TP and NCP volumes correlated with indices of ischemia and can predict future adverse cardiac events [50, 51]. Interestingly, CPV was associated with FFR on a per-vessel level, though not on a per-lesion level. This discrepancy may, in part, be attributed to the influence of a single vessel-specific study included in our meta-analysis [32], which contributed to a large number of vessels and applied a notably lower CP threshold (> 150 HU), potentially capturing a broader range of plaque misclassified as calcified, hence the larger volume differences. Furthermore, the lack of calcium scoring in that study complicated the evaluation of whether the lower threshold in the particular study merely overestimated CPV or effectively identified low-density calcifications. Generally, CPV is less associated with ischemia compared to lipid-rich plaques, as calcified lesions often represent stable atherosclerotic lesions that do not impair coronary flow [52]. The association between plaque volumes and FFR may have been weakened by the different attenuation cut-off limits used across the included studies. However, defining precise attenuation thresholds for distinguishing between NCP and CP remains challenging, as they have been shown to vary substantially with the degree of contrast enhancement in the coronary lumen (e.g., iodine concentration), tube potential, and reconstruction kernels [53].

High-risk plaque characteristics, including LAP, NRS, PR, and SC along with greater PB, have previously been linked to elevated risk of myocardial infarction and death [9, 54–56]. A previous study utilizing IVUS and FFR demonstrated a strong correlation between lipid-rich plaques and reduced FFR values [57]. LAP, considered a CCTA surrogate of necrotic core, was notably larger in ischemia-causing lesions [58, 59], and given the association between NRS, PR, SC, and lipid-rich content, their alignment with LAP findings was anticipated [60]. Consequently, the meta-analysis revealed that the presence of LAP, NRS, and SC plaques was significantly associated with an increased probability of ischemia at both per-lesion and per-vessel levels.

While luminal stenosis is a recognized predictor of hemodynamically significant FFR, its correlation with FFR in patients with stable CAD is imperfect, especially in intermediate coronary lesions [5, 61]. This anatomical-physiological mismatch suggests that plaque composition and burden may contribute to ischemia independently of luminal stenosis. The present analysis expands on these insights by demonstrating that incorporating both quantitative and qualitative plaque characteristics enhances ischemia prediction. Despite the variability in AUC values across the included studies, %APV outperformed luminal stenosis alone in one study [17] and showed higher AUCs when combined with luminal stenosis in three studies [17, 39, 42]. Furthermore, the addition of %APV to models integrating stenosis severity and qualitative features enhanced discrimination, reinforcing the value of combining anatomical and compositional plaque assessments for ischemia prediction in two studies [39, 42].

Although the current findings were largely consistent across studies, the variability from 2010 to 2024 in CT hardware (temporal and spatial resolution), CT acquisition settings, and post-processing algorithms may have influenced our findings and undoubtedly impacted image quality and plaque quantification results. While several included studies utilized software from the same vendor, version differences may have affected result consistency. A prior CT study by Mancini et al found high plaque correlation within a single analysis platform, but reduced reliability across platforms, regardless of adaptive or fixed attenuation thresholds [62]. Similarly, interscan reproducibility of plaque quantification was lower when different CT vendors were utilized compared to using the same vendor [63].

The findings of this meta-analysis have important clinical and technological implications for understanding and managing patients with CAD. Incorporating both quantitative and qualitative plaque characteristics into diagnostic workflows may potentially improve ischemia prediction, offering better risk stratification and guidance for treatment. Standardization of CT acquisition practice and plaque quantification methods, e.g., by establishing uniform thresholds for plaque components and consensus definitions for qualitative features is crucial. Such steps may facilitate more robust comparisons across studies and ensure reproducibility of results in clinical and research practice. Advances in automated plaque analysis software and radiomics hold significant potential to enhance reproducibility and diagnostic accuracy by integrating plaque composition and burden and other CT imaging-derived features [64, 65], while the incorporation of artificial intelligence and machine learning algorithms into plaque analysis may further improve prediction of ischemia by identifying patterns in large datasets that are not discernible through manual analysis. In addition to coronary plaque quantification, emerging evidence highlights pericoronary fat attenuation, derived from CCTA, as a novel biomarker linked to coronary inflammation and ischemia [66]. Its integration with plaque characteristics could enhance ischemia prediction and refine patient risk stratification. Future research should focus on a standardized exploration in an integrated fashion of various CT-derived plaque, inflammation, and physiological metrics for potentially improved and individualized patient management.

### Limitations

Although this meta-analysis of over 4400 patients undergoing CCTA and FFR provided significant insights into the association between coronary plaque characteristics and ischemia, several limitations must be acknowledged, consequently, the results should be interpreted cautiously. Most of the included studies were of retrospective design, and patients were highly selected as they all underwent ICA and FFR, which may have introduced selection bias. The variability in CT scanner types and vendors along with different plaque assessment tools with differing levels of automation across the studies may have impacted the consistency of imaging protocols, reconstruction algorithms, and plaque quantification techniques, and may have affected the comparability of results. Furthermore, the differing definitions and thresholds for plaques, particularly for NCP and CP varied substantially between studies that may have caused some heterogeneity in plaque data combination. Converting medians with ranges to means with standard deviations in a few studies may have introduced some inaccuracies. Additionally, the heterogeneity in reported AUC data precluded their eligibility for pooling in the meta-analysis. We were not able, from this dataset, to perform individual or adjusted data analysis. These factors collectively may constrain the generalizability of the findings and emphasize the need for standardized methodologies and prospective validation to strengthen the clinical relevance.

## Conclusion

This meta-analysis highlights that quantitatively and qualitatively CCTA-derived plaque characteristics are significantly associated with ischemia, as assessed by invasively measured FFR. Moreover, incorporating detailed plaque characteristics improved the discrimination of ischemia compared to stenosis evaluation alone. Prospective studies are needed to validate these findings and translate them into improved management strategies for patients with CAD.

## Supplementary information

ELECTRONIC SUPPLEMENTARY MATERIAL

## Abbreviations

APV: Aggregate plaque volume
CCTA: Coronary computed tomography angiography
CP: Calcified plaque
LAP: Low attenuation plaque
LL: Lesion length
NCP: Non-calcified plaque
NRS: Napkin-ring sign
PB: Plaque burden
PR: Positive remodeling
RI: Remodeling index
SC: Spotty calcification
