# Supplementary material for: Influence of plaque characteristics by coronary computed tomography angiography on lesion-specific ischemia: a systematic review and meta-analysis
Source: Eur Radiol. 2025 Mar 27;35(10):6271–90. doi: 10.1007/s00330-025-11516-1 (PMC12417243; doi:10.1007/s00330-025-11516-1)
Supplement: Supplementary file 1 — ELECTRONIC SUPPLEMENTARY MATERIAL [file 330_2025_11516_MOESM1_ESM.pdf]

Influence of plaque characteristics by coronary computed tomography angiography on lesion-specific ischemia. A systematic review and meta-analysis.

ELECTRONIC SUPPLEMENTARY MATERIAL

TABLES

Table S1. Excluded studies and reason for exclusion

Table S2. Search strategy of electronic databases.

Table S3. Quality Assessment of Diagnostic Accuracy Studies (QUADAS-2) score for diagnostic studies.

Table S4. The PRISMA Checklist

FIGURES

Figure S1. Meta-analysis of quantitative plaque parameters comparing lesions with FFR ≤0.80 versus >0.80.

Figure S2. Meta-analysis of quantitative plaque parameters comparing vessels with FFR ≤0.80 versus >0.8

Table S1. Excluded studies and reason for exclusion

**Ineligible criteria or data**
1. Zhang, C., et al., Prognostic Value of Gai's Plaque Score and Agatston Coronary Artery Calcium Score for Functionally Significant Coronary Artery Stenosis. Chin Med J (Engl), 2016. 129(23): p. 2792-2796.
2. Imai, S., et al., Abnormal fractional flow reserve in nonobstructive coronary artery disease: The relationship with plaque characteristics. Circulation: Cardiovascular Interventions, 2019. **12**(2).
3. Baumann, S., et al., Coronary CT angiography derived plaque markers correlated with invasive instantaneous flow reserve for detecting hemodynamically significant coronary stenoses. Eur J Radiol, 2020. **122**: p. 108744.
4. Aoshima, C., et al., Plaque characteristics on coronary CT angiography associated with the positive findings of fractional flow reserve and instantaneous wave-free ratio. Heart Vessels, 2021. **36**(4): p. 461-471.
5. Varga-Szemes, A., et al., Coronary plaque assessment of Vasodilative capacity by CT angiography effectively estimates fractional flow reserve. Int J Cardiol, 2021. **331**: p. 307-315.
6. Yan, H., et al., Pericoronary fat attenuation index and coronary plaque quantified from coronary computed tomography angiography identify ischemia-causing lesions. Int J Cardiol, 2022. **357**: p. 8-13.
7. Liu, M., et al., Predictive value of DEEPVESSEL-fractional flow reserve and quantitative plaque analysis based on coronary CT angiography for major adverse cardiac events. Clin Radiol, 2023. **78**(9): p. e600-e607.
8. Zhou, K., et al., Incremental diagnostic value of radiomics signature of pericoronary adipose tissue for detecting functional myocardial ischemia: a multicenter study. Eur Radiol, 2023. **33**(5): p. 3007-3019.
**Insufficient data**
1. Min, J.K., et al., Coronary CTA plaque volume severity stages according to invasive coronary angiography and FFR. J Cardiovasc Comput Tomogr, 2022. **16**(5): p. 415-422.
2. Han, D., et al., Sex differences in computed tomography angiography-derived coronary plaque burden in relation to invasive fractional flow reserve. J Cardiovasc Comput Tomogr, 2023. **17**(2): p. 112-119.
**Double publication**
1. Diaz-Zamudio, M., et al., Automated Quantitative Plaque Burden from Coronary CT Angiography Noninvasively Predicts Hemodynamic Significance by using Fractional Flow Reserve in Intermediate Coronary Lesions. Radiology, 2015. **276**(2): p. 408-15.

2. Ghekiere, O., et al., Diagnostic performance of quantitative coronary computed tomography angiography and quantitative coronary angiography to predict hemodynamic significance of intermediate-grade stenoses. Int J Cardiovasc Imaging, 2015. **31**(8): p. 1651-61.

3. Nakazato, R., et al., Additive diagnostic value of atherosclerotic plaque characteristics to non-invasive FFR for identification of lesions causing ischaemia: Results from a prospective international multicentre trial. EuroIntervention, 2016. **12**(4): p. 473-481.

4. Tesche, C., et al., Coronary CT angiography derived morphological and functional quantitative plaque markers correlated with invasive fractional flow reserve for detecting hemodynamically significant stenosis. J Cardiovasc Comput Tomogr, 2016. **10**(3): p. 199-206.

5. Baskaran, L., et al., Dense calcium and lesion-specific ischemia: A comparison of CCTA with fractional flow reserve. Atherosclerosis, 2017. **260**: p. 163-168.

6. Ahmadi, A., et al., Lesion-Specific and Vessel-Related Determinants of Fractional Flow Reserve Beyond Coronary Artery Stenosis. JACC Cardiovasc Imaging, 2018. **11**(4): p. 521-530.

7. Dey, D., et al., Integrated prediction of lesion-specific ischaemia from quantitative coronary CT angiography using machine learning: a multicentre study. Eur Radiol, 2018. **28**(6): p. 2655-2664.

8. Øvrehus, K.A., et al., CT-based total vessel plaque analyses improves prediction of hemodynamic significance lesions as assessed by fractional flow reserve in patients with stable angina pectoris. J Cardiovasc Comput Tomogr, 2018. **12**(4): p. 344-349.

9. Yu, M., et al., Relationship of the Duke jeopardy score combined with minimal lumen diameter as assessed by computed tomography angiography to the hemodynamic relevance of coronary artery stenosis. J Cardiovasc Comput Tomogr, 2018. **12**(3): p. 247-254.

10. Yu, M., et al., Diagnostic performance of perivascular fat attenuation index to predict hemodynamic significance of coronary stenosis: a preliminary coronary computed tomography angiography study. Eur Radiol, 2020. **30**(2): p. 673-681.

11. Bom, M.J., et al., Diagnostic value of comprehensive on-site and off-site coronary CT angiography for identifying hemodynamically obstructive coronary artery disease. J Cardiovasc Comput Tomogr, 2021. **15**(1): p. 37-45.

12. Yang, S., et al., Prognostic Implications of Comprehensive Whole Vessel Plaque Quantification Using Coronary Computed Tomography Angiography. JACC: Asia, 2021. **1**(1): p. 37-48.

13. Brandt, V., et al., Additive value of epicardial adipose tissue quantification to coronary CT angiography-derived plaque characterization and CT fractional flow reserve for the prediction of lesion-specific ischemia. Eur Radiol, 2022. **32**(6): p. 4243-4252.

14. Lin, A., et al., Machine Learning From Quantitative Coronary Computed Tomography Angiography Predicts Fractional Flow Reserve-Defined Ischemia and Impaired Myocardial Blood Flow. Circ Cardiovasc Imaging, 2022. **15**(10): p. e014369

Table S2. Search strategy of electronic databases.

Database	Search strategy and syntaxes	Number of retrieved articles<br>PubMed/Embase/Cochrane
PubMed (Medline) / EMBASE/Cochrane	**Search: computed tomography coronary plaque fractional flow reserve**<br>**Filters: English, from 2005-2024**<br>(("tomography, x ray computed"[MeSH Terms] OR ("tomography"[All Fields] AND "x ray"[All Fields] AND "computed"[All Fields]) OR "x-ray computed tomography"[All Fields] OR ("computed"[All Fields] AND "tomography"[All Fields]) OR "computed tomography"[All Fields]) AND ("coronaries"[All Fields] OR "heart"[MeSH Terms] OR "heart"[All Fields] OR "coronary"[All Fields]) AND ("plaque s"[All Fields] OR "plaque, amyloid"[MeSH Terms] OR ("plaque"[All Fields] AND "amyloid"[All Fields]) OR "amyloid plaque"[All Fields] OR "plaque"[All Fields] OR "dental plaque"[MeSH Terms] OR ("dental"[All Fields] AND "plaque"[All Fields]) OR "dental plaque"[All Fields] OR "plaques"[All Fields]) AND "fractional"[All Fields] AND ("flow camb"[Journal] OR "flow"[All Fields]) AND ("reserve"[All Fields] OR "reserve s"[All Fields] OR "reserves"[All Fields])) AND ((english[Filter]) AND (2010:2023[pdat]))	**230 /229/20**
	**Search: computed tomography coronary plaque ischemia flow**<br>**Filters**: **English, from 2005-2024**<br>(("tomography, x ray computed"[MeSH Terms] OR ("tomography"[All Fields] AND "x ray"[All Fields] AND "computed"[All Fields]) OR "x-ray computed tomography"[All Fields] OR ("computed"[All Fields] AND "tomography"[All Fields]) OR "computed tomography"[All Fields]) AND ("coronaries"[All Fields] OR "heart"[MeSH Terms] OR "heart"[All Fields] OR "coronary"[All Fields]) AND ("plaque s"[All Fields] OR "plaque, amyloid"[MeSH Terms] OR ("plaque"[All Fields] AND "amyloid"[All Fields])	**137/152/15**

	OR "amyloid plaque"[All Fields] OR "plaque"[All Fields] OR "dental plaque"[MeSH Terms] OR ("dental"[All Fields] AND "plaque"[All Fields]) OR "dental plaque"[All Fields] OR "plaques"[All Fields]) AND ("ischaemia"[All Fields] OR "ischemia"[MeSH Terms] OR "ischemia"[All Fields] OR "ischaemias"[All Fields] OR "ischemias"[All Fields]) AND ("flow camb"[Journal] OR "flow"[All Fields])) AND ((english[Filter]) AND (2010:2023[pdat]))	
	**Search: computed tomography coronary plaque characteristics fractional flow reserve**<br>**Filters: English, from 2005-2024**<br>(("tomography, x ray computed"[MeSH Terms] OR ("tomography"[All Fields] AND "x ray"[All Fields] AND "computed"[All Fields]) OR "x-ray computed tomography"[All Fields] OR ("computed"[All Fields] AND "tomography"[All Fields]) OR "computed tomography"[All Fields]) AND ("coronaries"[All Fields] OR "heart"[MeSH Terms] OR "heart"[All Fields] OR "coronary"[All Fields]) AND ("plaque s"[All Fields] OR "plaque, amyloid"[MeSH Terms] OR ("plaque"[All Fields] AND "amyloid"[All Fields]) OR "amyloid plaque"[All Fields] OR "plaque"[All Fields] OR "dental plaque"[MeSH Terms] OR ("dental"[All Fields] AND "plaque"[All Fields]) OR "dental plaque"[All Fields] OR "plaques"[All Fields]) AND ("characteristic"[All Fields] OR "characteristics"[All Fields]) AND "fractional"[All Fields] AND ("flow camb"[Journal] OR "flow"[All Fields]) AND ("reserve"[All Fields] OR "reserve s"[All Fields] OR "reserves"[All Fields])) AND (english[Filter])	**103/92/6**
	**Search: computed tomography coronary plaque features fractional flow reserve**<br>**Filters: English, from 2005-2024**<br>(("tomography, x ray computed"[MeSH Terms] OR ("tomography"[All Fields] AND "x ray"[All Fields] AND "computed"[All Fields]) OR "x-ray computed tomography"[All Fields] OR ("computed"[All Fields] AND	**41 /35/4**

	"tomography"[All Fields]) OR "computed tomography"[All Fields]) AND ("coronaries"[All Fields] OR "heart"[MeSH Terms] OR "heart"[All Fields] OR "coronary"[All Fields]) AND ("plaque s"[All Fields] OR "plaque, amyloid"[MeSH Terms] OR ("plaque"[All Fields] AND "amyloid"[All Fields]) OR "amyloid plaque"[All Fields] OR "plaque"[All Fields] OR "dental plaque"[MeSH Terms] OR ("dental"[All Fields] AND "plaque"[All Fields]) OR "dental plaque"[All Fields] OR "plaques"[All Fields]) AND ("feature s"[All Fields] OR "featured"[All Fields] OR "features"[All Fields] OR "featuring"[All Fields] OR "protein domains"[MeSH Terms] OR ("protein"[All Fields] AND "domains"[All Fields]) OR "protein domains"[All Fields] OR "feature"[All Fields]) AND "fractional"[All Fields] AND ("flow camb"[Journal] OR "flow"[All Fields]) AND ("reserve"[All Fields] OR "reserve s"[All Fields] OR "reserves"[All Fields])) AND (english[Filter])	
**Total results of PubMed/EMBASE**		**511/508/45**
**Total results of all electronic search**		**1064**
**Duplicates**		**683**
**Number of retrieved results for evaluation**		**381**

Table S3. Quality Assessment of Diagnostic Accuracy Studies (QUADAS-2) score for diagnostic studies.

Study author and publication year	Risk of bias				Applicability		
	Patient selection	Index test	Reference standard	Flow and timing	Patient selection	Index test	Reference standard
**Lesion-specific studies**							
Kristensen 2010	L	L	L	L	L	L	L
Nakazato 2013	L	L	L	L	L	L	L
Li 2013	L	L	L	L	L	L	L
Rossi 2014	U	L	L	L	L	L	L
Doh 2014	L	L	L	L	U	L	L
Opolski 2014	U	L	L	L	L	L	L
Park 2015	L	L	L	L	L	L	L
Hell 2015	L	L	L	L	L	L	L
Wang 2015	L	L	L	L	L	L	L
Gaur 2016	L	L	L	L	L	L	L
Zhang 2018	H	L	L	H	L	L	L
Yu 2019	H	L	L	L	L	L	L
Doeberitz 2019	L	L	L	L	L	L	L
Du 2020	L	U	U	L	L	U	L
Li 2021	U	L	L	L	L	L	L
Yang 2021	L	U	L	L	U	U	L
Lee 2024	L	L	L	L	L	L	L
Long 2024	L	L	U	L	L	U	H
**Vessel-specific studies**							
Rizvi 2017	L	L	L	L	L	L	L
Driessen 2018	L	L	L	L	L	L	L

Lee 2019	L	L	L	L	L	L	L
Kawai 2021	U	L	L	L	L	L	L
Yin 2021	L	L	L	U	L	L	L
Zhao 2021	L	L	L	L	L	L	L
Ma 2021	L	L	L	L	L	L	L
Velangi 2021	L	L	L	L	L	L	L
Tang 2022	H	L	L	L	L	L	L
Yan 2023	L	L	L	L	L	L	L
Lee 2023	L	L	L	L	L	L	L
Wang 2023	L	L	L	L	L	L	L
**Lesion- and vessel-specific study**							
Kato 2017	U	L	L	U	L	L	L
**Total**	3H+4U+24L	2U+29L	2U+29L	1H+2U+28L	2U+29L	3U+28L	1H+30L

Abbreviations: H=high, L=low, U=unclear.

Table S4. The PRISMA checklist

Section and Topic	Item #	Checklist item	Location where item is reported
**TITLE**			
Title	1	Identify the report as a systematic review.	p. 1
**ABSTRACT**			
Abstract	2	See the PRISMA 2020 for Abstracts checklist.	p. 3
**INTRODUCTION**			
Rationale	3	Describe the rationale for the review in the context of existing knowledge.	p. 5
Objectives	4	Provide an explicit statement of the objective(s) or question(s) the review addresses.	p. 5
**METHODS**			
Eligibility criteria	5	Specify the inclusion and exclusion criteria for the review and how studies were grouped for the syntheses.	p. 6
Information sources	6	Specify all databases, registers, websites, organisations, reference lists and other sources searched or consulted to identify studies. Specify the date when each source was last searched or consulted.	p. 5-6
Search strategy	7	Present the full search strategies for all databases, registers and websites, including any filters and limits used.	p. 5-6, supp p. 4-6
Selection process	8	Specify the methods used to decide whether a study met the inclusion criteria of the review, including how many reviewers screened each record and each report retrieved, whether they worked independently, and if applicable, details of automation tools used in the process.	p. 6
Data collection process	9	Specify the methods used to collect data from reports, including how many reviewers collected data from each report, whether they worked independently, any processes for obtaining or confirming data from study investigators, and if applicable, details of automation tools used in the process.	p. 6-7
Data items	10a	List and define all outcomes for which data were sought. Specify whether all results that were compatible with each outcome domain in each study were sought (e.g. for all measures, time points, analyses), and if not, the methods used to decide which results to collect.	p. 6-7
	10b	List and define all other variables for which data were sought (e.g. participant and intervention characteristics, funding sources). Describe any assumptions made about any missing or unclear information.	p. 6-7
Study risk of bias assessment	11	Specify the methods used to assess risk of bias in the included studies, including details of the tool(s) used, how many reviewers assessed each study and whether they worked independently, and if applicable, details of automation tools used in the process.	p. 7
Effect measures	12	Specify for each outcome the effect measure(s) (e.g. risk ratio, mean difference) used in the synthesis or presentation of results.	p. 8
Synthesis methods	13a	Describe the processes used to decide which studies were eligible for each synthesis (e.g. tabulating the study intervention characteristics and comparing against the planned groups for each synthesis (item #5)).	p. 8
	13b	Describe any methods required to prepare the data for presentation or synthesis, such as handling of missing summary statistics, or data conversions.	p. 8
	13c	Describe any methods used to tabulate or visually display results of individual studies and syntheses.	p. 8

Section and Topic	Item #	Checklist item	Location where item is reported
	13d	Describe any methods used to synthesize results and provide a rationale for the choice(s). If meta-analysis was performed, describe the model(s), method(s) to identify the presence and extent of statistical heterogeneity, and software package(s) used.	p. 8
	13e	Describe any methods used to explore possible causes of heterogeneity among study results (e.g. subgroup analysis, meta-regression).	
	13f	Describe any sensitivity analyses conducted to assess robustness of the synthesized results.	
Reporting bias assessment	14	Describe any methods used to assess risk of bias due to missing results in a synthesis (arising from reporting biases).	p. 8
Certainty assessment	15	Describe any methods used to assess certainty (or confidence) in the body of evidence for an outcome.	p. 8
**RESULTS**			
Study selection	16a	Describe the results of the search and selection process, from the number of records identified in the search to the number of studies included in the review, ideally using a flow diagram.	p.8<br>supp p. 2-3<br>Figure 2
	16b	Cite studies that might appear to meet the inclusion criteria, but which were excluded, and explain why they were excluded.	Sup p. 2-3
Study characteristics	17	Cite each included study and present its characteristics.	Table 1
Risk of bias in studies	18	Present assessments of risk of bias for each included study.	Sup p. 8
Results of individual studies	19	For all outcomes, present, for each study: (a) summary statistics for each group (where appropriate) and (b) an effect estimate and its precision (e.g. confidence/credible interval), ideally using structured tables or plots.	p. 9-11<br>Figure 3-6
Results of syntheses	20a	For each synthesis, briefly summarise the characteristics and risk of bias among contributing studies.	p. 11
	20b	Present results of all statistical syntheses conducted. If meta-analysis was done, present for each the summary estimate and its precision (e.g. confidence/credible interval) and measures of statistical heterogeneity. If comparing groups, describe the direction of the effect.	p. 10-11<br>Figure 3-6
	20c	Present results of all investigations of possible causes of heterogeneity among study results.	p. 10-11<br>Figure 3-6
	20d	Present results of all sensitivity analyses conducted to assess the robustness of the synthesized results.	p. 10-11<br>Figure 3-6
Reporting biases	21	Present assessments of risk of bias due to missing results (arising from reporting biases) for each synthesis assessed.	p. 11
Certainty of evidence	22	Present assessments of certainty (or confidence) in the body of evidence for each outcome assessed.	p. 10-11<br>Figure 3-6
**DISCUSSION**			

Section and Topic	Item #	Checklist item	Location where item is reported
Discussion	23a	Provide a general interpretation of the results in the context of other evidence.	p. 12-15
	23b	Discuss any limitations of the evidence included in the review.	p. 14-16
	23c	Discuss any limitations of the review processes used.	p. 15-16
	23d	Discuss implications of the results for practice, policy, and future research.	p. 14-15
**OTHER INFORMATION**			
Registration and protocol	24a	Provide registration information for the review, including register name and registration number, or state that the review was not registered.	p. 5
	24b	Indicate where the review protocol can be accessed, or state that a protocol was not prepared.	p. 5
	24c	Describe and explain any amendments to information provided at registration or in the protocol.	
Support	25	Describe sources of financial or non-financial support for the review, and the role of the funders or sponsors in the review.	p. 1
Competing interests	26	Declare any competing interests of review authors.	p. 1
Availability of data, code and other materials	27	Report which of the following are publicly available and where they can be found: template data collection forms; data extracted from included studies; data used for all analyses; analytic code; any other materials used in the review.	

From: Page MJ, McKenzie JE, Bossuyt PM, Boutron I, Hoffmann TC, Mulrow CD, et al. The PRISMA 2020 statement: an updated guideline for reporting systematic reviews. BMJ 2021;372:n71. doi: 10.1136/bmj.n71. This work is licensed under CC BY 4.0. To view a copy of this license, visit https://creativecommons.org/licenses/by/4.0/

Figure S1. Meta-analysis of quantitative plaque parameters comparing lesions with FFR >0.80 versus ≤0.80.

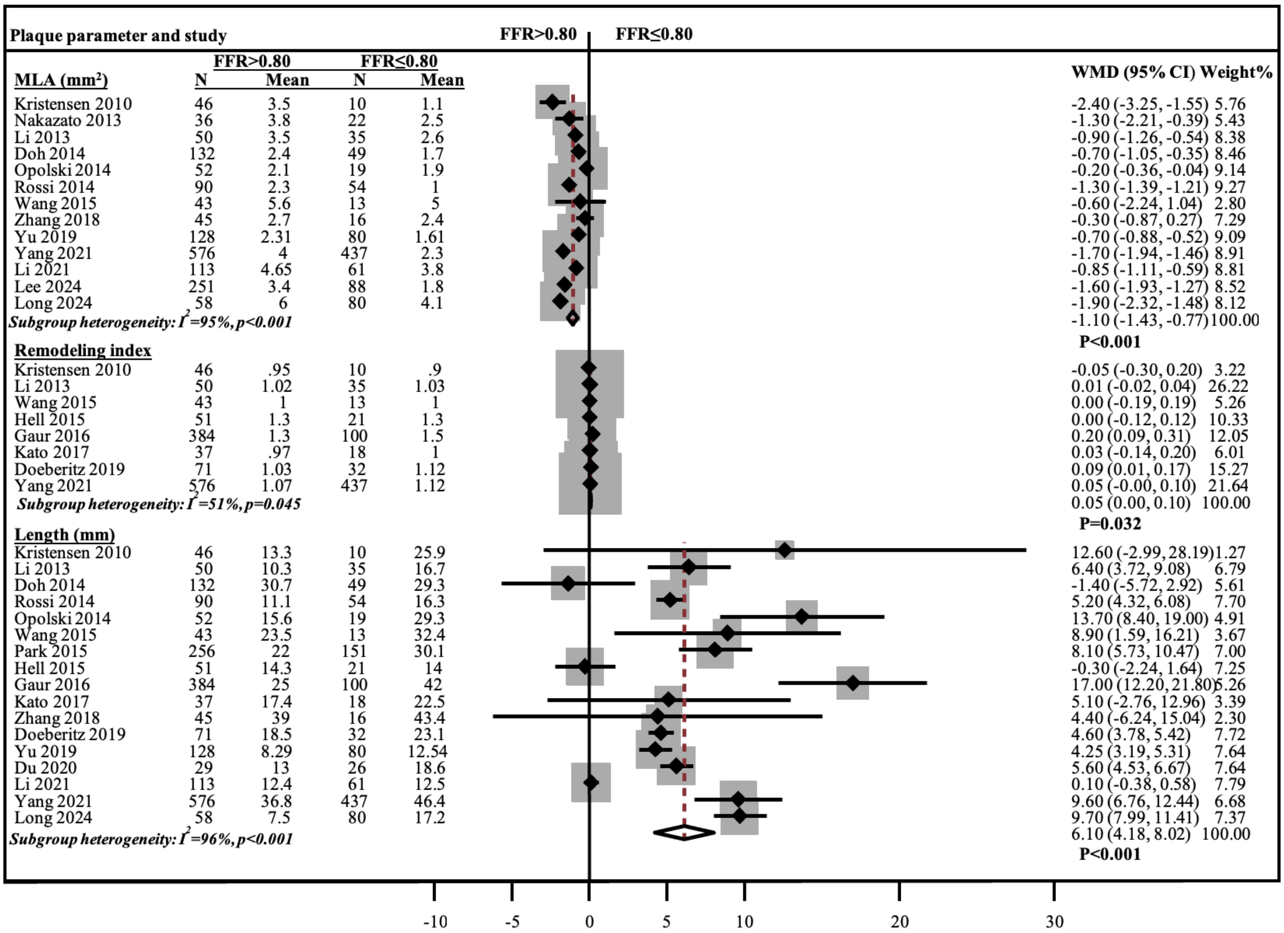

Abbreviations: CI=confidence interval, FFR=fractional flow reserve, MLA=minimal lumen area, N=number, RI=remodeling index, WMD=weighted mean difference.

Figure S2. Meta-analysis of quantitative plaque parameters comparing vessels with FFR >0.8 versus ≤0.80.

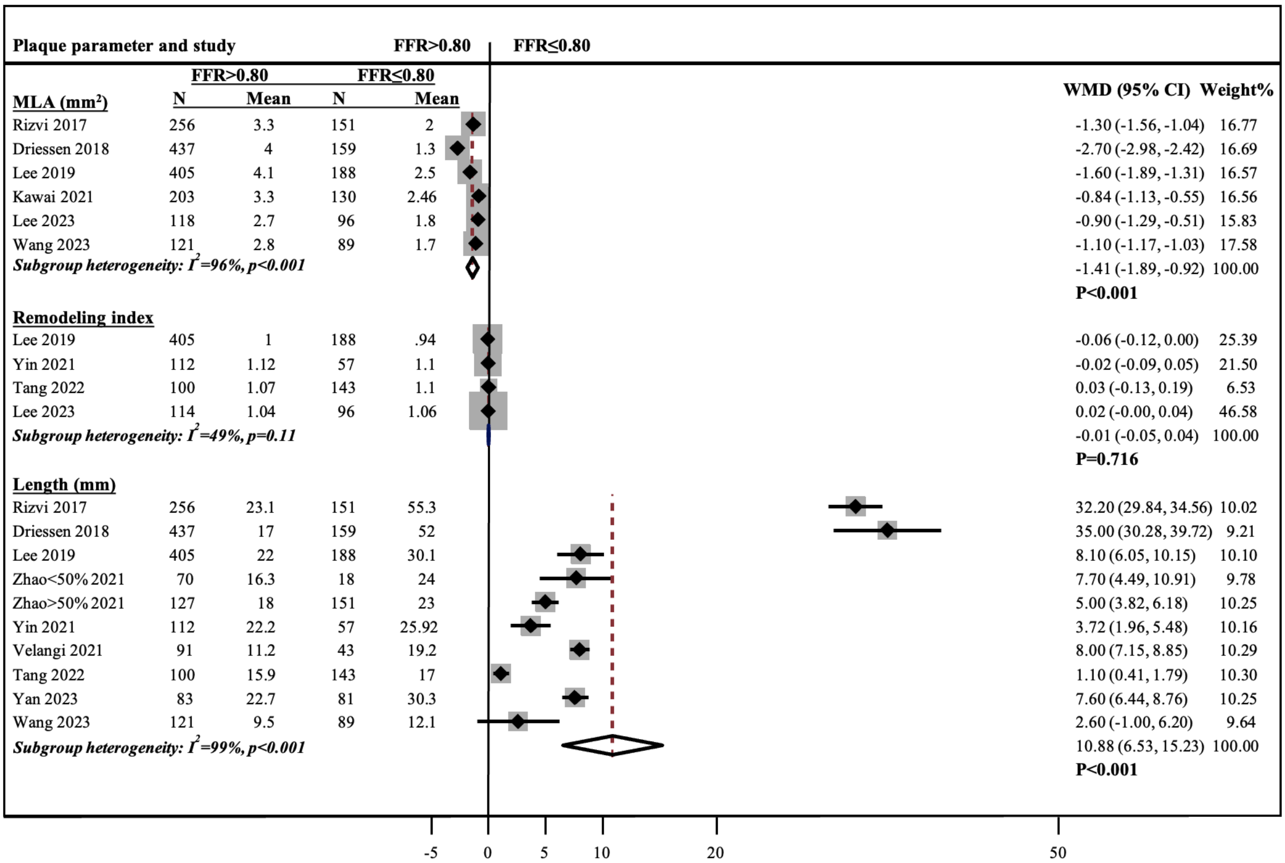

Abbreviations: CI=confidence interval, FFR=fractional flow reserve, MLA=minimal lumen area, N=number, RI=remodeling index, WMD=weighted mean difference.
